# Proceedings of the Medical Society of the County of Herkimer

**Published:** 1897-11

**Authors:** 


### Society Proceedings.

### PROCEEDINGS OF THE MEDICAL SOCIETY OF THE COUNTY OF HERKIMER.

A SPECIAL meeting of the Medical Society of the County of Herkimer was held at the Spellman House, at Middleville, Saturday, October 9th, at 10 A. M., having been called for the purpose of taking appropriate action upon the death of Dr. C. W. Hamlin, of Middleville.

The president, Dr. W. E. HAYS, of Frankfort, took the chair and called the meeting to order.

Dr. A. WALTER SUITER announced to the society the death of Dr. Hamlin and proceeded to make remarks concerning his admirable qualities and particularly referred, at some length and with much feeling, to his intimate personal and professional relations with the deceased. He then presented the following minute and moved its unanimous adoption by the society, and that the secretary be directed to inscribe the same upon the records of the meeting.

Dr. SUITER's motion was seconded by Dr. D. M. DEVENDORF and carried.

On motion of Dr. S. R. MILLINGTON, of Poland, the secretary was also directed to secure the publication of the record.

### MEMORIAL OF CHARLES WILLARD HAMLIN, M. D.

The Medical Society of the County of Herkimer, in special meeting assembled, having learned with extreme regret and sorrow of the death of our late distinguished fellow-member and associate, Charles Willard Hamlin, M. D., would hereby spread upon our minutes a record of his lamented demise and of our highest appreciation of him as a man, a physician, a soldier, a citizen and a friend.

He was born at Holland Patent, N. Y., December 21, 1839. He was graduated at an early age from the Whitestown Seminary and finished his literary courses of study by graduation from the State Normal School, at Albany, N. Y. He chose the profession of medicine and began its study in his native town under the office tutelage of Drs. D. A. Crane and Norton Wolcott. Shortly thereafter he heard the call "to arms!" and nobly responded by sacrificing his personal prospects to his country's needs, and enrolled himself as a private soldier in the 57th regiment of New York volunteer infantry. His antecedents as a medical student and his aptitude for hospital service at once won for him the position of hospital steward, and he was assigned to duty from time to time in both field and general hospitals during the entire three years for which he was enlisted. In this relation his associations were of the highest and most profitable character, in both an executive and a professional sense, and qualified him most admirably for his subsequent studies which he pursued, after his honorable discharge from the army, in the Bellevue Hospital Medical College, of New York City, whence he was graduated as a doctor of medicine in March, 1866. He began the practice of medicine at once in Oriskany Falls, N. Y., where he remained for a few years, when he came to this county and succeeded to the practice of his father-in-law, Dr. A. E. Darney, of Middleville, who was for many years an able and distinguished member of this society.

Dr. Hamlin was made a member of this society June 2, 1871, and has been an active and prominent figure herein since that time. He has been chosen to all the principal offices within the gift of the society, and has officiated in committee work with unexceled judgment and skill. As chairman for many years of the standing committee on hygiene he was especially active, and his able reports were written most intelligently, and some of them were honored by incorporation in the records of the corresponding committee of the state society.

He wrote many valuable papers, and his productions were characterised by great literary merit and originality. He was notable for the prominent part he always took in the discussion of general subjects, both medical and surgical, and constantly proved his thorough familiarity with the progress of every department of science. He represented this society for four years as a delegate to the Medical Society of the State of New York and was made a permanent member of that body by special election in 1890. His interest in the state society's progress was manifested by the fact that he attended as a spectator and invited guest for fifteen years before he received his regular delegateship.

As a citizen he was noted for his liberal public spirit, and served the public interest most faithfully and intelligently in various positions of trust and responsibility.

His death took place at St. Luke's Hospital, in the City of Utica, on the morning of the 7th of October, 1897, and was caused by diabetic gangrene.

The doctor was the eldest of a family of eleven children and was the first to die. He is survived by his wife and one son, Dr. Varney B. Hamlin, of Clinton, N. Y. His father is still living in Holland Patent, N. Y., at the age of eighty-eight years. The funeral was held from the Church of the Memorial, Middleville, Saturday morning, October 9th, and, following the example set by his daughter, his remains were taken to Waterville, N. Y., and there cremated.

Dr. Hamlin's information was broad, his understanding was clear, his perception acute. He was frank and sincere in his utterances, generous in his every impulse, modest in his bearing. He loved nature, of which he was a most consistent student. He was devoted to his work as a physician and never failed to respond to the cry of distress, however onerous was the burden he was called upon to bear. In the monotonous grind of the daily duties of the medical practitioner, the details of which are oftentimes so painfully familiar to us all, he demonstrated on all occasions the great moral courage and devoted unselfishness of which he was so abundantly possessed. We take pardonable pride in offering the record of his worth and his self-sacrificing deeds for emulation. In his death we feel that we have lost a most valuable professional associate and a consistent, honorable and amiable friend.

He was for many years physician to the County House and County Insane Asylum before it passed to state control. He was a member of the Pan-American Medical Congress, and was one of those selected to represent the State of New York in that great organisation in Washington in 1893. He was a member of the New York Physicians' Mutual Aid Association of New York City and a member of the Protestant Episcopal Memorial Church, of Middleville. He was a broad-minded physician, enjoying the patronage of a wide and populous district. He was an obstetrician of superior qualification, an expert diagnostician and had comprehensive and far-seeing judgment as to prognosis and treatment. He was universally regarded, wherever known, as an "all-around" medical practitioner, both in attending and consulting service. He was a member of the Grand Army of the Republic and a Free Mason of high degree, having attained to the title of the thirty-second degree or Ancient Scottish Rite. Soon after the death of his only daughter, a beautiful and accomplished young woman, he traveled extensively in Europe, and while climbing the Alps unfortunately met with an accident, thereby producing a wound on his great toe which failed to heal kindly, and was a subject of much concern to him during his sojourn abroad. Very serious symptoms rapidly developed after reaching home, necessitating a resort to amputation.

In propriâ personâ our friend and fellow-member now disappears from the sight of men. And while we bespeak for his ashes peace, they are left to remind us of the precious memory of

Life's duties done,
Life's race well run,
Life's victories well won,
Now cometh rest.

We extend to his stricken family and his immediate friends our profound sympathy, and beg that they will permit us to unite in grief with all who mourn.

We shall meet, but we shall miss him;
There will be one vacant chair.

The Society then adjourned and proceeded to attend the funeral in a body.

